# Cost-effectiveness of zoledronic acid compared with sequential denosumab/alendronate for older osteoporotic women in Japan

**DOI:** 10.1007/s11657-021-00956-z

**Published:** 2021-07-15

**Authors:** Takahiro Mori, Carolyn J. Crandall, Tomoko Fujii, David A. Ganz

**Affiliations:** 1 Department of General Medical Science, Graduate School of Medicine, Chiba University, 1-8-1 Inohana, Chuo-ku, Chiba, Chiba 260-8670 Japan; 2 Health Services Research and Development Center, University of Tsukuba, Tsukuba, Ibaraki Japan; 3 Department of General Internal Medicine, Eastern Chiba Medical Center, Togane, Chiba Japan; 4 Division of General Internal Medicine and Health Services Research, Department of Medicine, David Geffen School of Medicine at University of California, Los Angeles, Los Angeles, CA USA; 5 Department of Medical Research and Management for Musculoskeletal Pain, 22nd Century Medical and Research Center, University of Tokyo Hospital, Tokyo, Japan; 6 Geriatric Research, Education and Clinical Center and HSR&D Center for the Study of Healthcare Innovation, Implementation and Policy, Veterans Affairs Greater Los Angeles Healthcare System, Los Angeles, CA USA; 7 Division of Geriatrics, Department of Medicine, David Geffen School of Medicine at University of California, Los Angeles, Los Angeles, CA USA; 8 Health Unit, RAND Corporation, Santa Monica, CA USA

**Keywords:** Cost-effectiveness analysis, Osteoporosis, Fracture prevention, Zoledronic acid, Denosumab

## Abstract

***Summary*:**

Among hypothetical cohorts of older osteoporotic women without prior fragility fracture in Japan, we evaluated the cost-effectiveness of two treatment strategies using a simulation model. Annual intravenous zoledronic acid for 3 years was cost-saving compared with biannual subcutaneous denosumab for 3 years followed by weekly oral alendronate for 3 years.

**Purpose:**

Osteoporosis constitutes a major medical and health economic burden to society worldwide. Injectable treatments for osteoporosis require less frequent administration than oral treatments and therefore have higher persistence and adherence with treatment, which could explain better efficacy for fracture prevention. Although annual intravenous zoledronic acid and biannual subcutaneous denosumab are available, it remains unclear which treatment strategy represents a better value from a health economic perspective. Accordingly, we examined the cost-effectiveness of zoledronic acid for 3 years compared with sequential denosumab/alendronate (i.e., denosumab for 3 years followed by oral weekly alendronate for 3 years, making the total treatment duration 6 years) among hypothetical cohorts of community-dwelling osteoporotic women without prior fragility fracture in Japan at ages 65, 70, 75, or 80 years.

**Methods:**

Using a previously validated and updated Markov microsimulation model, we obtained incremental cost-effectiveness ratios (Japanese yen [¥] (or US dollars [$]) per quality-adjusted life-year [QALY]) from the public healthcare and long-term care payer’s perspective over a lifetime horizon with a willingness-to-pay of ¥5 million (or $47,500) per QALY.

**Results:**

In the base case, zoledronic acid was cost-saving (i.e., more effective and less expensive) compared with sequential denosumab/alendronate. In deterministic sensitivity analyses, results were sensitive to changes in the efficacy of zoledronic acid or the cumulative persistence rate with zoledronic acid or denosumab. In probabilistic sensitivity analyses, the probabilities of zoledronic acid being cost-effective were 98–100%.

**Conclusions:**

Among older osteoporotic women without prior fragility fracture in Japan, zoledronic acid was cost-saving compared with sequential denosumab/alendronate.

**Supplementary Information:**

The online version contains supplementary material available at 10.1007/s11657-021-00956-z.

## Introduction

Osteoporosis constitutes a major burden to society worldwide, and pharmacologic therapy to prevent fractures is the mainstay of treatment. Persistence and adherence with medications are key factors influencing the efficacy of pharmacologic therapy, where persistence refers to “the duration of time from initiation to discontinuation of the therapy” and adherence refers to “the extent to which a patient acts in accordance with the prescribed interval and dose of a dosing regimen” [1].

Injectable treatments for osteoporosis require less frequent administration than oral treatments. As a result, persistence and adherence are more favorable with injectable medications and could explain better efficacy for fracture prevention. This better persistence and adherence associated with less frequent administration may offset the higher cost per dose for injectable medications. This was the case in our previous health economic evaluation, which compared subcutaneous denosumab given every 6 months (the least frequent dose of a medication available for the treatment of osteoporosis in Japan at the time of the analysis) with oral alendronate given weekly [2]. In hypothetical cohorts of older women with osteoporosis without prior hip or vertebral fracture in Japan, we found that denosumab was cost-effective (i.e., more effective but more expensive, and the incremental cost-effectiveness ratio (ICER) is less than the predetermined threshold of willingness-to-pay) or cost-saving (i.e., more effective and less expensive) mainly due to denosumab’s higher persistence rate leading to better efficacy for fracture prevention compared with oral alendronate.

In this previous work, we did not include a subsequent treatment after the completion of denosumab [2]. However, recent studies have demonstrated that those treated with denosumab should not have a drug holiday after a given treatment period (in contrast to those treated with bisphosphonates), because discontinuation of denosumab increases the risk of vertebral fractures [3–5]. Therefore, it is recommended that a subsequent treatment, typically bisphosphonates, be prescribed after the completion of denosumab [6]. To the best of our knowledge, however, no cost-effectiveness analysis regarding fracture prevention has been reported worldwide that includes a strategy in which denosumab was followed by a subsequent treatment.

Intravenous zoledronic acid annually was not included in our previous study, as it had been approved for some other indications but not for the treatment of osteoporosis in Japan at the time of the analysis [2]. Zoledronic acid for the treatment of osteoporosis has been approved since September 2016 in Japan, and currently, annual zoledronic acid and biannual denosumab are the two treatments for osteoporosis with the longest dosing intervals. However, it remains unknown which treatment strategy represents better value from a health economic perspective: zoledronic acid or denosumab followed by bisphosphonates.

As a typical and realistic scenario for the treatment of osteoporotic women, we examined the cost-effectiveness of intravenous zoledronic acid annually for 3 years compared with sequential denosumab/alendronate, which we defined as subcutaneous denosumab every 6 months for 3 years (a period matching that of zoledronic acid) followed by weekly oral alendronate for 3 years, making the total duration of treatment 6 years [6, 7]. Since these treatments’ optimal durations have not been determined, we explored the effect of longer durations of treatment in sensitivity analyses, exploring up to 6 years of use for zoledronic acid, or up to 10 years’ use of denosumab or alendronate [6–10].

## Materials and methods

### Overview

We updated a Markov microsimulation model validated in previous work [2, 11–13] to perform a cost-effectiveness analysis among hypothetical cohorts of community-dwelling osteoporotic women in Japan without prior fragility fracture, at various ages of therapy initiation (65, 70, 75, and 80 years). We estimated quality-adjusted life-years (QALYs) and total costs in 2020 Japanese yen (¥). For ease of interpretation, we converted these results to US dollars ($) at a rate of ¥105 to $1, which approximates the current exchange rate as of December 2020 [14]. We obtained ICERs, representing cost per QALY gained for one strategy compared with the others, over a lifetime horizon (until a participant reached age 105 years, or died).

Japan developed a universal healthcare insurance system in 1961, and separately launched a mandatory public long-term care insurance system in 2000. Those age 65 and older are eligible for long-term care services, including not only institutional care (e.g., long-term admission or short-term stay in a long-term care facility) but also community- and home-based care (e.g., adult day care, outpatient rehabilitation, home help, or home-visit nursing) [15]. Thus, for the base case, we evaluated cost-effectiveness from the public healthcare and long-term care payer’s perspective (i.e., the perspective of a single payer responsible for both public healthcare costs and long-term care costs) [13]. The public healthcare payer’s perspective (including public healthcare costs, but not including long-term care costs) was adopted as a sub-analysis (Table 1).

**Table 1:** Impact inventory

Type of Impact	Perspective	
	Public healthcare and long-term care payer	Public healthcare payer
Formal healthcare sector		
Health outcomes (effects)		
Longevity	☑	☑
Health-related quality-of-life	☑	☑
Other (e.g., adverse events)	☑	☑
Medical costs		
Medications	☑	☑
Physician visits	☑	☑
Blood tests	☑	☑
DXA scans	☑	☑
Future related costs (i.e., treatment for fractures)	☑	☑
Future unrelated medical costs	☐	☐
Non−healthcare sector		
Cost of long-term care after fracture	☑	☐
Cost of unpaid lost productivity due to fracture	☐	☐

The willingness-to-pay threshold was set to ¥5 million ($47,500) per QALY in the base case [2]. In deterministic and probabilistic sensitivity analyses, we also evaluated a willingness-to-pay threshold of ¥10 million ($95,000) per QALY [13, 16]. We discounted all costs and health benefits at 2% per year for the base case [17]. This study’s reporting followed the Consolidated Health Economic Evaluation Reporting Standards (CHEERS) statement and recommendations for the conduct of economic evaluation in osteoporosis [18, 19] (Supplemental Tables 1, 2).

We performed an extensive systematic review of all the parameters in the model (Table 2). Inputs were derived from peer-reviewed literature (e.g., meta-analyses of randomized controlled trials or observational studies, individual observational studies, and cost-effectiveness analyses) and websites (e.g., statistics reports from the Ministry of Health, Labour and Welfare, drug prices, and currency exchange rate) that were considered the most relevant (e.g., Japanese population), high-quality, and up-to-date estimates. We used our own assumptions only if no reliable published estimate was available. We used TreeAge Pro Healthcare 2020 (TreeAge Software Inc., Williamstown, MA, USA) to program the model.

**Table 2:** Model parameters

	Value (base case)	Range for deterministic sensitivity analysis	Distribution and range for probabilistic sensitivity analysis	Reference
Efficacy (relative risk)				
Zoledronic acid for hip fracture	0.64	0.47–0.86^*^	Beta: 0.47–0.86^*^	[21]
Zoledronic acid for clinical vertebral fracture	0.40	0.29–0.55^*^	Beta: 0.29–0.55^*^	
Denosumab for hip fracture	0.56	0.31–0.94^*^	Beta: 0.31–0.94^*^	
Denosumab for clinical vertebral fracture	0.30	0.21–0.43^*^	Beta: 0.21–0.43^*^	
Alendronate for hip fracture	0.64	0.45–0.88^*^	Beta: 0.45–0.88^*^	
Alendronate for clinical vertebral fracture	0.50	0.40–0.64^*^	Beta: 0.40–0.64^*^	
Cumulative persistence rates, first, second, and third year (%)				
Zoledronic acid	100, 52, 36	100, 40–65, 23–50^*^ 3)^+^ 100, 87, 85^*^ (ages 65, 70, 75) 3)^+^ 100, 67, 57^*^ (age 80)	Beta: 100, 40–65, 23–50^*^	[22]
Denosumab	81, 55, 37	1)^+^ 76–85, 48–63, 33–43^*#^ 2)^+^, 3)^+^ 94, 92, 87^*^ (age 65, 70, 75) 2)^+^, 3)^+^ 83, 71, 59^*^ (age 80)	Beta: 76–85, 48–63, 33–43^*#^	[22, 23]
Alendronate	55, 39, 28	Triangular: ± 25%^#^	Triangular: ± 25%^#^	[24]
Adherence rates at first, second, and third year (%)				N/A
Zoledronic acid	100, 100, 100	N/A	N/A	N/A
Denosumab	100, 100, 100	N/A	N/A	[22]
Alendronate	71, 68, 66	Triangular: ± 13%^#^	Triangular: ± 13%^#^	[24]
Costs ¥ (U.S. dollars), ¥ 105 = 1 U.S. dollars				
Annual medication costs and costs for prescription charge at pharmacy				
Zoledronic acid	¥38,000 ($360)	N/A	N/A	[38]
Denosumab	¥58,000 ($550)	60% of the current cost^#^	N/A	
Alendronate	¥8700 ($83)	N/A	N/A	
Prescription charge for alendronate	¥1700 ($16)	N/A	N/A	[39]
Costs for physician visit, blood test, and DXA scan				
First visit, zoledronic acid	¥5500 ($52)	N/A	N/A	[39]
Subsequent visit, zoledronic acid	¥3900 ($37)	N/A	N/A	
First visit, denosumab	¥3100 ($30)	N/A	N/A	
Subsequent visit, denosumab	¥1500 ($14)	N/A	N/A	
First visit, alendronate	¥3600 ($34)	N/A	N/A	
Subsequent visit, alendronate	¥1900 ($18)	N/A	N/A	
Blood test	¥2900 ($28)	N/A	N/A	
DXA scan	¥4500 ($43)	N/A	N/A	
Medical costs				
Hip fracture	¥1,726,000 ($16,440)	± 50%^#^	Triangular: ± 50%^#^	[40]
First clinical vertebral fracture	¥420,000 ($4000)	± 50%^#^	Triangular: ± 50%^#^	
Subsequent clinical vertebral fracture	¥842,000 ($8020)			
Annual long-term care costs				
The “post-hip fracture” state	¥876,000 ($8340)	± 50%^#^	Triangular: ± 50%^#^	[40, 42]
The “post-vertebral fracture” state	¥213,000 ($2030)			
Utilities				
Ages 65–69	0.862	N/A	Triangular: ± 15%^#^	[32]
Ages 70–74	0.810	N/A		
Ages 75–79	0.771	N/A		
Ages 80–84	0.769	N/A		
Age 85 +	0.684	N/A		
Disutilities (multiplier)				
Hip fracture, first year	0.776	N/A	Beta: 0.720–0.844^*^	[33, 34]
Hip fracture, beyond first year	0.855	N/A	Beta: 0.800–0.909^*^	
Clinical vertebral fracture, first year	0.724	N/A	Beta: 0.667–0.779^*^	
Clinical vertebral fracture, beyond first year	0.868	N/A	Beta: 0.827–0.922^*^	
Annual incidence rates of hip fracture per 100,000 persons (without intervention**)**				
Age 65–69	83.9	± 50%^#^	Triangular: ± 10%^#^	[26]
Age 70–74	158.1			
Age 75–79	362.2			
Age 80–84	851.1			
Age 85–89	1580.2			
Age 90–94	2466.0			
Age 95–99	2961.7			
Age 100 +	2471.0			
Annual incidence rates of clinical vertebral fracture per 100,000 persons (without intervention)				
Age 65–69	156.7	± 50%^#^	Triangular ± 25%^#^	[26, 27]
Age 70–74	513.9			
Age 75–79	1106.2			
Age 80–84	2034.1			
Age 85–89	2331.2			
Age 90–95	3638.0			
Age 95–100	4369.3			
Age 100 +	3645.4			
Relative risks of hip fracture for individuals with osteoporosis				
Age 65–69	2.39	N/A	Gamma: 2.16–2.60^*^	[2]
Age 70–74	1.89	N/A	Gamma: 1.79–1.99^*^	
Age 75–79	1.57	N/A	Gamma: 1.52–1.62^*^	
Age 80–84	1.35	N/A	Gamma: 1.32–1.38^*^	
Age 85 +	1.25	N/A	Gamma: 1.22–1.27^*^	
Relative risks of clinical vertebral fracture for individuals with osteoporosis				
Age 65–69	2.47	N/A	Gamma: 2.10–2.86^*^	[2]
Age 70–79	2.09	N/A	Gamma: 1.84–2.34^*^	
Age 80 +	1.86	N/A	Gamma: 1.68–2.04^*^	
Relative risks of subsequent fracture associated with prior fracture at the same location				
Hip fracture	2.3	N/A	Gamma: 1.5–3.7^*^	[2]
Clinical vertebral fracture	4.4	N/A	Gamma: 3.6–5.4^*^	
Relative hazards for mortality after a hip fracture				
Within a year	2.87	N/A	Gamma: 2.52–3.27^*^	[30]
Second year and beyond	1.73	N/A	Gamma: 1.56–1.90^*^	
Relative hazards for mortality after a clinical vertebral fracture				
Within a year	1.0	2.87^#^ (same as hip fracture)	N/A	[13]
Second year and beyond	1.0	1.73^#^ (same as hip fracture)	N/A	
Proportions of excess mortality attributable to a fracture (%)				
Hip fracture	25	N/A	Triangular: 0–50^#^	[2]
Clinical vertebral fracture	0	25^#^ (same as hip fracture)	N/A	
Discount rates (%)				
Costs	2	N/A	Triangular: 0–4^@^	[17]
Quality-adjusted life-years	2	N/A	Triangular: 0–4^@^	

1)^+^ Scenario based on the upper bound of the 95% credible interval of a meta-analysis and our own assumption[21]

2)^+^ Scenario based on a small retrospective observational study at a single institution in Japan (*n* = 102)[23]

3)^+^ Scenario where in addition to higher cumulative persistence rates of denosumab based on a small observational study in Japan, higher cumulative persistence rates of zoledronic acid were modeled, assuming that the same ratios of the cumulative persistence rates of zoledronic acid to denosumab based on a meta-analysis were applied[21, 23]

^*^95% confidence or credible intervals based on literature

^#^Based on our own assumptions

^@^Based on the Official Guideline for the Economic Evaluation of Drugs/Medical Devices in Japan

### Model structure (Fig. 1)

**Fig. 1:**
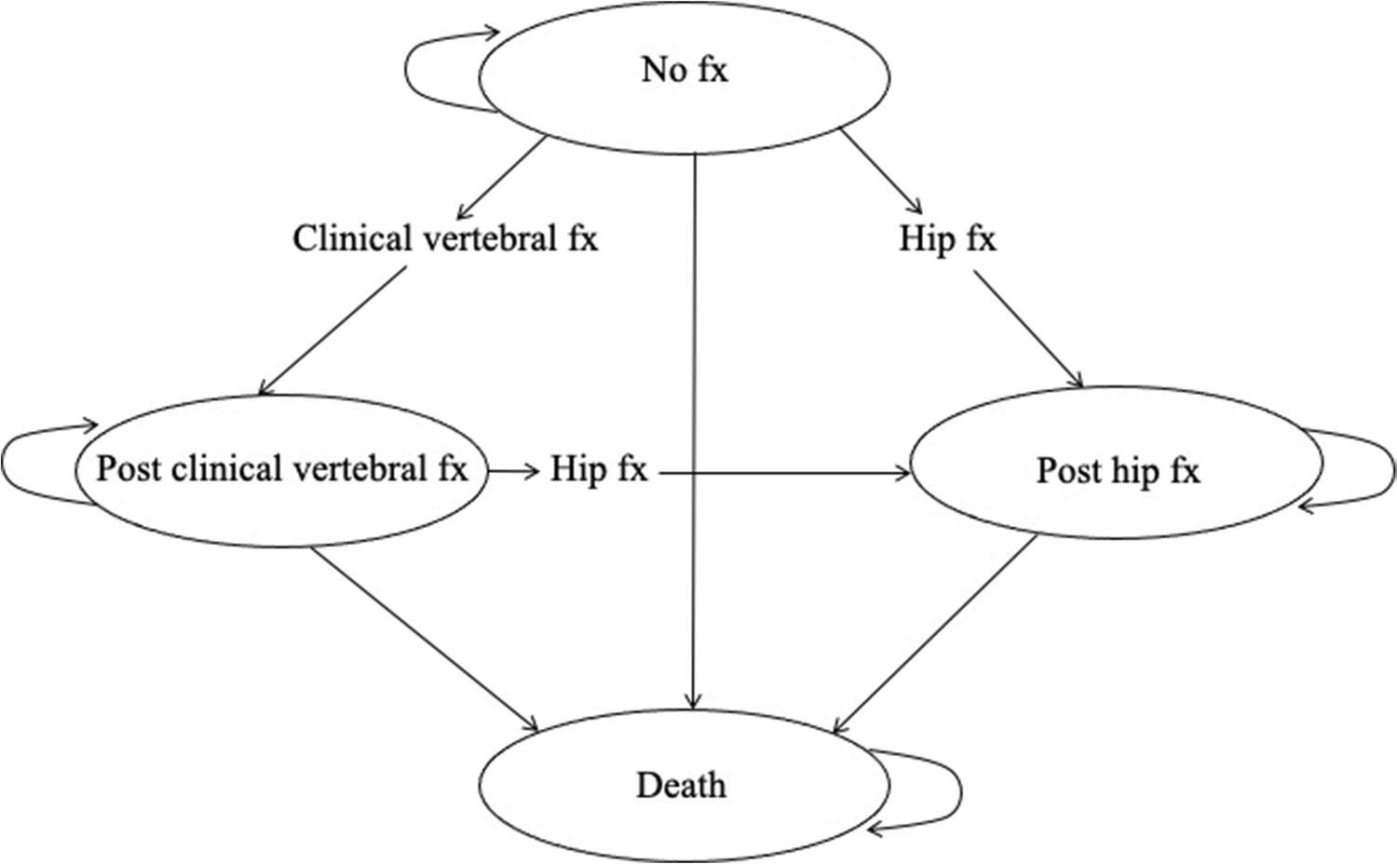
Markov diagram of health states and possible transitions. Every participant starts the model in the “no fracture” state, and transitions between the health states or remains in the same state based on the assigned transition probabilities between four Markov states, including no fracture, post-hip fracture, post-clinical vertebral fracture, and death. If a participant in the “post-hip fracture” state sustains a subsequent clinical vertebral fracture, the participant experiences a one-time cost and disutility associated with the clinical vertebral fracture, but the individual remains in the “post-hip fracture” state, as the “post-hip fracture” state incurs higher disutility beyond the first year than the “post-clinical vertebral fracture” state in this model

Each cycle lasts 1 year, and every participant may sustain a hip or clinical vertebral fracture during each cycle. A participant can sustain only one fracture per cycle and can have a maximum of two hip fractures and an unlimited number of clinical vertebral fractures over the entire time horizon. We used tracker variables for treatments and fractures to incorporate memory of previous events in each individual from one cycle to the next in the model [2, 11–13].

### Target population

The target population was postmenopausal osteoporotic women in Japan without prior fragility fracture. Consistent with the Japanese guidelines, osteoporosis was defined as a T-score ≤  − 2.5, or bone mineral density (BMD) ≤ 70% of the young adult mean (YAM), for the lumbar spine or hip (either femoral neck or total hip) as measured by dual-energy X-ray absorptiometry (DXA) [20]. In the model, individuals had already received a DXA scan and had been diagnosed with osteoporosis based on the DXA scan result.

### Efficacy of treatments

We compared the cost-effectiveness of intravenous zoledronic acid annually for 3 years compared with sequential denosumab/alendronate (i.e., subcutaneous denosumab every 6 months for 3 years followed by weekly oral alendronate for 3 years). Data from a recent systematic review and network meta-analysis of randomized controlled trials were used to obtain the efficacy of zoledronic acid, denosumab, and alendronate compared with placebo in reducing the risks of fragility fractures for those with osteoporosis [21]. Persistence rates with zoledronic acid and denosumab were based on meta-analyses of observational studies [22]. These meta-analyses showed that the cumulative persistence rates with zoledronic acid with a permissive gap of 90 days at the second dose (i.e., getting at 12 months, lasting till 24 months) and the third dose (i.e., getting at 24 months, lasting till 36 months) were 52 and 36%, respectively. As zoledronic acid is given once yearly, we assumed that the persistence rate with zoledronic acid was 100% at the end of the first year. The same study showed that, allowing for a permissive gap of 60 days, the cumulative persistence rates with denosumab at the second dose (i.e., getting at 6 months, lasting till 12 months) at the fourth dose (i.e., getting at 18 months, lasting till 24 months) and at the sixth dose (i.e., getting at 30 months, lasting till 36 months) were 81, 55, and 26%, respectively. The cumulative persistence rate at the second and fourth doses was based on a meta-analysis of 14 and 9 studies, respectively. The rate at the sixth dose was, however, based on a single study performed in Czech Republic rather than a meta-analysis of multiple studies, which raises the possibility that the result is not generalizable. Therefore, we conservatively assumed the same persistence rate from the second dose to the fourth dose applied to the persistence rate from the fourth dose to the sixth dose, making the rate at the sixth dose 37% (the 95% CI for this latter rate was estimated based on the 95% CI at the second year) [22].

A small retrospective observational study at a single institution in Japan (*n* = 102) showed cumulative persistence rates with denosumab of 94 (year 1), 92 (year 2), and 87% (year 3) for ages 65, 70, and 75, and 83 (year 1), 71 (year 2), and 59% (year 3) at age 80 [23]. This Japanese study was published after the meta-analysis had been conducted and was not included in the meta-analysis of the persistence rates of denosumab above [22]. As this small single-center study seemed to lack generalizability and we also could not find a counterpart study that examined the cumulative persistence rates of zoledronic acid in the Japanese setting, we evaluated these higher cumulative persistence rates of denosumab in a deterministic sensitivity analysis. In an additional deterministic sensitivity analysis, higher cumulative persistence rates of zoledronic acid were included in addition to the higher cumulative persistence rates of denosumab based on this study, assuming that the same ratios of the cumulative persistence rates of zoledronic acid to denosumab based on the previously noted meta-analysis were applied [22]. Those persistent with zoledronic acid or denosumab with the permissive gaps above were by definition also adherent to the medication.

The cumulative persistence rates with weekly bisphosphonates were estimated to be approximately 55, 39, and 28% at the end of the first, second, or third year, respectively, with a permissive gap of 30 days, and the adherence rates with weekly bisphosphonates were estimated to be 70.6 and 60.9% in the first and fifth year, respectively [24]. We assumed a linear decline in the adherence rates between the first and fifth year. Adherence rates with oral bisphosphonates were higher in clinical trials (mostly greater than 80%, as high as 100%) than observational studies that reflected actual clinical settings [25]. We estimated the relative effectiveness of alendronate in the community by assuming a linear relationship between relative risk reduction and adherence [2, 11–13].

We assumed that zoledronic acid and alendronate had efficacy from the first year through the end of the duration of the treatment (i.e., 3 years) and the risk for fractures after completing therapy returned to rates in the absence of the treatment after the same number of years as the treatment was given, in a gradual linear fashion (i.e., offset effects were assumed to be proportional to the treatment periods’ length) [2, 11–13]. Accumulating evidence has shown that after discontinuing denosumab, there is an increased risk of vertebral fractures within a relatively short period compared with those who continued denosumab [3–5]. Therefore, we assumed that the therapeutic effect of denosumab wore off rapidly at the end of the cycle when one received the final dose, and returned to the baseline risk without treatment. To keep the model parsimonious, we assumed that each individual obtained benefits of fracture prevention if she persisted in taking the treatment at the end of each cycle (i.e., 1 year). Those who were not persistent with denosumab did not start on alendronate after they stopped taking denosumab in this model.

### 5) Transition probabilities

#### a) Fracture rates

We only modeled the annual incidence rates of hip and clinical vertebral fractures, because reliable epidemiological data regarding other osteoporotic fractures are limited in Japan [13, 26, 27]. We did not model the risk for atypical femur fractures in this study, as atypical femur fractures are an extremely rare complication of bisphosphonates, especially for those using bisphosphonates for up to 5 years [6, 28]. We included the relative risks of fractures for individuals with osteoporosis compared with the general population and increased relative risks of second and subsequent fractures associated with prior fractures at the same location [2, 11].

#### b) Mortality rates

Mortality rates were obtained from the abridged 2018 life table [29]. Excess mortality rates after a hip fracture in the short term (within a year) and the long term (starting in the second year and continuing lifelong) were included [2, 11–13, 30]. We conservatively assumed that hip fracture events only contribute to 25% of the excess mortality, as comorbidities appear to play a large role. We did not assume excess mortality associated with clinical vertebral fractures [2, 11–13, 31]. However, in a sensitivity analysis, we assumed the same excess mortality associated with clinical vertebral fractures as with hip fractures [13].

### 6) Utilities

We used the EuroQol five dimension scale (EQ-5D) and assumed that disutilities (i.e., losses in health-related quality of life) associated with hip and clinical vertebral fractures were highest in the year immediately following the fracture, but persisted for the rest of life [32–34].

### 7) Costs

We divided costs into formal healthcare sector and non-healthcare sector costs and provided an impact inventory (Table 1) [35]. We assumed that costs were identical regardless of age.

#### a) Formal healthcare sector

We included the costs (the sums of payments by third-party payers and patients out-of-pocket) of medications, physician visits, prescription charges at a pharmacy, blood tests, DXA scans, and medical treatments after fractures.

In Japan, the Ministry of Health, Labour and Welfare determines drug price standards under Japan’s universal healthcare insurance system, including the prices of brand, biosimilar, and generic drugs [36]. Biosimilar drugs are biological products that are highly similar to the approved biologic reference products and have no clinically meaningful differences from the reference products [37]. A generic or biosimilar version of zoledronic acid or denosumab for osteoporosis treatment was not available in Japan at the time of this analysis, but a multicenter trial (including Japan) using biosimilar denosumab is ongoing (i.e., clinicaltrials.gov NCT03974100). We therefore modeled the hypothetical cost of biosimilar denosumab in a deterministic sensitivity analysis, in which we assumed that the annual cost of denosumab was 60% of the cost of the current brand product, while keeping efficacy the same. We based this estimate on the cost for biosimilar teriparatide when it became available in Japan in 2019 at 60% of the cost of the equivalent brand product [38]. The cost of alendronate was estimated based on the costs of generic alendronate. We charged the cost of 3 months’ supply (i.e., a single prescription filled) for those who discontinued alendronate within the first year. Similarly, we also charged the cost of one dose for those who stopped denosumab after the first dose. The costs of medications were proportional to persistence and adherence with the treatments.

Allowable charges based on the Japanese medical fee schedule for 2020 were used for the assumed costs of prescription charges at a pharmacy, physician visits (the cost incurred for the first visit was higher than that for subsequent visits), blood tests and the fees for interpreting the results, and DXA scans [39]. There is no solid consensus regarding when and how frequently to perform blood tests during treatment [20]. For those receiving zoledronic acid or denosumab, we assumed blood tests were performed pre- and post-injection. We, therefore, assumed that those who received zoledronic acid had physician visits and blood tests twice a year, that those who received denosumab had physician visits and blood tests four times a year, and that those who took alendronate had a physician visit four times a year and blood tests twice a year (a prescription of medications beyond 3 months is not allowed in Japan without an additional physician visit). In a sensitivity analysis, we assumed that those who received either zoledronic acid or denosumab had physician visits and blood tests twice a year. There also does not appear to be a consensus regarding when patients should undergo a DXA scan after the initiation of osteoporosis treatments [10, 12, 20]; we charged the costs of a DXA scan at the end of the third and the sixth year. For those receiving zoledronic acid, we included the costs of acetaminophen or nonsteroidal anti-inflammatory drugs (i.e., NSAIDs) for several days including prescription charges at a pharmacy as a part of the costs of physician visits for the potential side effect of flu-like symptoms (e.g., fever, myalgias, and arthralgias) [6].

We included medical resource use costs within 1 year after a fracture, including acute care and post-acute care as future related medical costs. The costs of treatment after hip and clinical vertebral fractures were based on a study using Japanese claims data [40]. Future unrelated medical costs were not considered in this analysis [12].

#### b) Non‑healthcare sector

Long-term care costs are considered non-healthcare sector costs in Japan [41]. We estimated the annual costs of long-term care post-hip fracture and post-clinical vertebral fracture, which were charged across all participants in the “post-hip fracture” state and the “post-clinical vertebral fracture” state until death [13, 42].

### Model simulation and sensitivity analysis

For base case analyses, we ran the model with 100,000 trials (100,000 individuals through the model one at a time). We performed deterministic (one-way) sensitivity analyses to evaluate the robustness of the results across a range of values for critical model parameters (Table 2). Although these treatments’ optimal durations have not been determined, those at high risk for osteoporotic fractures may benefit from up to 6 years of use of zoledronic acid, or up to 10 years’ use of denosumab or alendronate. We therefore performed a deterministic sensitivity analysis with longer durations of treatments (i.e., zoledronic acid for 6 years on, 2 years off, and 6 years on versus denosumab for 10 years followed by alendronate for 10 years). For this analysis, we assumed the same relative rate of persistence with zoledronic acid or denosumab in the fourth year and beyond as in the third year. This resulted in cumulative persistence rates of zoledronic acid decreasing to 25, 17, and 12% in the fourth, fifth, and sixth year, respectively, and cumulative persistence rates with denosumab decreasing to 25, 17, 12, 8, 5, 4, and 2% in the fourth through tenth years, respectively. We assumed that those who took alendronate for 7 years continued to take alendronate for up to 10 years (i.e., no dropout from eighth year onward except for death) with the same adherence rate as the fifth year from the sixth year onward [24]. We also performed two additional sensitivity analyses, in which (1) we assumed the same excess mortality associated with clinical vertebral fracture as with hip fracture, and (2) we assumed that those who received either zoledronic acid or denosumab had physician visits and blood tests twice a year.

Next, we performed probabilistic sensitivity analyses, in which parameter values were randomly selected from their probability distributions for uncertain key model inputs. Monte Carlo simulation was performed with 1000 simulations and 100,000 trials per simulation. To verify the model’s accuracy, we initially included a “no-intervention” arm in calculating mortality and fracture rates in the model.

## Results

### Model validation

Our model predicted that the probabilities of dying by age 105 with different starting ages (i.e., 65, 70, 75, or 80) were greater than 99%, consistent with the 2018 Japanese life table [29]. Our model also predicted that without treatment, the probabilities after the starting ages of having at least one hip fracture were 21% at ages 65 or 70, and 20% at ages 75 or 80, respectively. The probabilities of having at least one clinical vertebral fracture were 44% at age 65 or 70, 43% at age 75, and 39% at age 80, respectively.

### Base case analysis

Zoledronic acid was cost-saving (i.e., more effective and less expensive) compared with sequential denosumab/alendronate at all ages examined. Costs and effectiveness at various starting ages were as follows: age 65, $23,710 and 14.219 QALYs for zoledronic acid and $24,160 and 14.218 QALYs for denosumab/alendronate; age 70, $24,050 and 11.628 QALYs for zoledronic acid and $24,540 and 11.626 QALYs for denosumab/alendronate; age 75, $22,930 and 9.201 QALYs for zoledronic acid and $23,500 and 9.196 QALYs for denosumab/alendronate; and age 80, $19,650 and 6.942 QALYs for zoledronic acid and $20,290 and 6.935 QALYs for denosumab/alendronate. From the public healthcare payer’s perspective, the conclusions remained the same (Table 3).

**Table 3:** The results of the base case analyses at various ages of therapy initiation

	Lifetime cost (US dollars, $1 = ¥105)	Quality-adjusted life-years (QALY)	Incremental cost-effectiveness ratio
From the public healthcare and long-term care payer’s perspective (primary analysis)			
Age 65			
Zoledronic acid	$23,710	14.219	Cost-saving
Denosumab/alendronate	$24,160	14.218	Comparator
Age 70			
Zoledronic acid	$24,050	11.628	Cost-saving
Denosumab/alendronate	$24,540	11.626	Comparator
Age 75			
Zoledronic acid	$22,930	9.201	Cost-saving
Denosumab/alendronate	$23,500	9.196	Comparator
Age 80			
Zoledronic acid	$19,650	6.942	Cost-saving
Denosumab/alendronate	$20,290	6.935	Comparator
From the public healthcare payer’s perspective (sub-analysis)			
Age 65			
Zoledronic acid	$9320	14.219	Cost-saving
Denosumab/alendronate	$9760	14.218	Comparator
Age 70			
Zoledronic acid	$9810	11.628	Cost-saving
Denosumab/alendronate	$10,260	11.626	Comparator
Age 75			
Zoledronic acid	$9750	9.201	Cost-saving
Denosumab/alendronate	$10,240	9.196	Comparator
Age 80			
Zoledronic acid	$8810	6.942	Cost-saving
Denosumab/alendronate	$9340	6.935	Comparator

### Deterministic sensitivity analysis

**Fig. 2:**
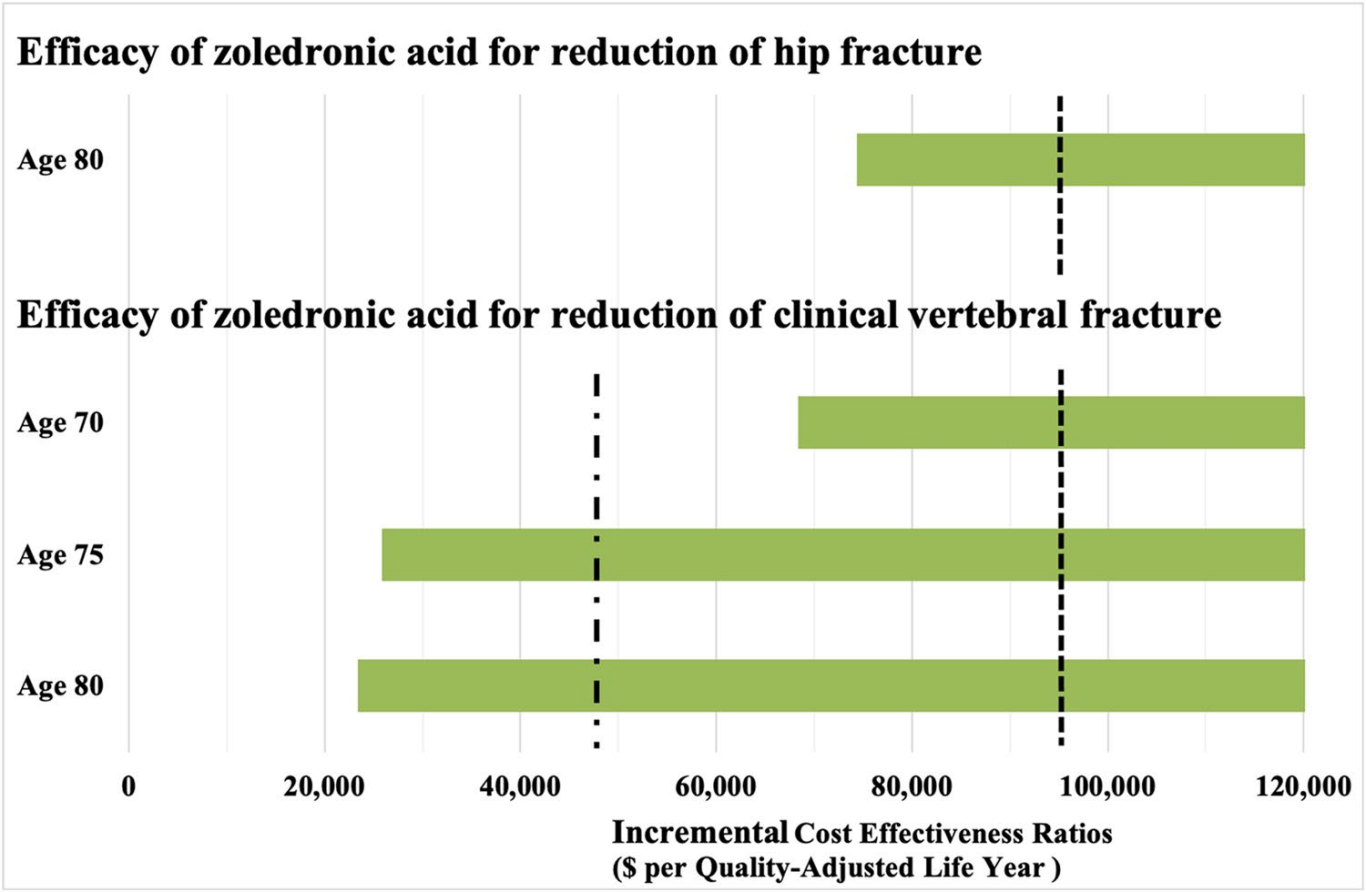
Results of deterministic sensitivity analyses. The figure presents the incremental cost-effectiveness ratios (ICERs) of zoledronic acid compared with sequential denosumab/alendronate at different starting ages, when the parameter estimates varied across its ranges of plausible values. The public healthcare and long-term care payer’s perspective is taken. The green bars represent the ranges of possible values in deterministic sensitivity analyses (cut off on right-hand side for ease of display). The vertical hashed line represents the predetermined thresholds of willingness-to-pay of $47,500 (¥5 million) or $95,000 (¥10 million) per quality-adjusted life-year. The ICERs of deterministic sensitivity analyses that became less than the predetermined thresholds of willingness-to-pay were presented

Results were sensitive to changes in the efficacy of zoledronic acid for reduction of clinical vertebral or hip fracture. The ICERs for denosumab/alendronate compared to zoledronic acid became less than the willingness-to-pay threshold of ¥5 million ($47,500) per QALY only at ages 75 and 80 with lower efficacy of zoledronic acid for reduction of clinical vertebral fracture (Fig. 2). Results were also sensitive to cumulative persistence rates (Table 4). If the cumulative persistence rates of denosumab were based on a small observational study in Japan (a rate higher than the upper value of the 95% CI of the meta-analysis results used for the base case), the ICERs became less than the willingness-to-pay threshold of 5million/QALY at ages 70 and 75. If we further assumed that the cumulative persistence rates of both zoledronic acid and denosumab were higher based on the Japanese study, the ICERs became greater than the willingness-to-pay threshold of ¥5 million/QALY at ages 70 and 75. Otherwise, in deterministic sensitivity analyses, zoledronic acid remained cost-saving compared with sequential denosumab, except that for some unfavorable values of zoledronic acid at age 65, zoledronic acid was not cost-saving, but denosumab/alendronate did not become cost-effective even at the willingness-to-pay threshold of ¥10 million/QALY. Finally, at all ages examined, zoledronic acid remained cost-saving with the specified sensitivity analyses for longer planned durations of treatment, assuming the same excess mortality associated with clinical vertebral fracture as with hip fracture, or assuming those who took either zoledronic acid or denosumab had physician visits and blood tests twice a year.

**Table 4:** The results of cost-effectiveness analysis with different assumptions of the cumulative persistence rates

	Cumulative persistence rates, first, second, and third year (%)		Age 65	Age 70	Age 75	Age 80
	Denosumab	ZA				
Base case (ages 65, 70, 75, or 80)	81, 55, 37	100, 52, 36	ZA: cost-saving	ZA: cost-saving	ZA: cost-saving	ZA: cost-saving
Sensitivity analysis 1^*^ (ages 65, 70, 75, or 80)	85, 63, 43	100, 52, 36	ICER of D/A $980,400/QALY	ICER of D/A $699,300/QALY	ICER of D/A $537,000/QALY	ZA: cost-saving
Sensitivity analysis 2^**^ (ages 65, 70, or 75)	94, 92, 87	100, 52, 36	ICER of D/A $87,200/QALY	ICER of D/A $29,000/QALY	ICER of D/A $6,400/QALY	N/A
Sensitivity analysis 3^***^ (ages 65, 70, or 75)	94, 92, 87	100, 87, 85	ICER of D/A $176,300/QALY	ICER of D/A $111,300/QALY	ICER of D/A $60,900/QALY	N/A
Sensitivity analysis 2^**^ (age 80)	83, 71, 59	100, 52, 36	N/A	N/A	N/A	ICER of D/A $50,500/QALY
Sensitivity analysis 3^***^ (age 80)	83, 71, 59	100, 67, 57	N/A	N/A	N/A	ZA: cost-saving

ZA, zoledronic acid; D/A, sequential denosumab/alendronate; ICER, incremental cost-effectiveness ratio; QALY, quality-adjusted life-years

The predetermined thresholds of willingness-to-pay were $47,500 (¥5 million) or $95,000 (¥10 million) per quality-adjusted life-year

^*^Scenario based on the upper bound of the 95% credible interval of a meta-analysis and our own assumption[21]

^**^Scenario based on a small retrospective observational study at a single institution in Japan (*n* = 102)[23]

^***^Scenario where in addition to higher cumulative persistence rates of denosumab based on a small observational study in Japan, higher cumulative persistence rates of zoledronic acid were modeled, assuming that the same ratios of the cumulative persistence rates of zoledronic acid to denosumab based on a meta-analysis were applied[21, 23]

### Probabilistic sensitivity analysis

The probabilities of zoledronic acid being cost-effective were 100% for ages 65 and 70, and 98% for ages 75 and 80, respectively, at a willingness-to-pay threshold of ¥5 million ($47,500) per QALY. The probabilities were 100% for age 65, 99% for age 70, and 97% for ages 75 and 80, respectively, at a willingness-to-pay threshold of ¥10 million ($95,000) per QALY.

## Discussion

We examined the cost-effectiveness of annual intravenous zoledronic acid for 3 years compared with sequential biannual subcutaneous denosumab for 3 years followed by weekly oral alendronate for 3 years among hypothetical cohorts of community-dwelling older osteoporotic women in Japan without prior fragility fracture. In our model, without treatment, the lifetime probabilities of a woman having a hip or vertebral fracture were 20–21 or 39–44%, respectively, representing a high-risk population for osteoporotic fracture. Zoledronic acid was cost-saving compared with sequential denosumab/alendronate at all ages examined.

We examined 3 years of planned treatment for zoledronic acid, a typical treatment period, followed by a drug holiday for 3 years. The treatment period for denosumab was matched to the period of zoledronic acid, followed by 3 years of alendronate. These treatments’ optimal durations, however, have not been determined. As a sensitivity analysis, therefore, we examined longer planned durations of treatment and found zoledronic acid remained cost-saving. Since at the end of the third year, the cumulative persistence rates of zoledronic acid and denosumab were only 36 and 37%, respectively, the extension of the treatments to only a small portion of the cohorts beyond 3 years did not influence the results.

Zoledronic acid was cost-saving despite the combined direct and residual effect of zoledronic acid (6 years) being shorter than sequential denosumab/alendronate (9 years, a 3-year effect of denosumab followed by a 6-year direct and residual effect of alendronate) due to several factors. First, zoledronic acid had an offset effect (i.e., the residual effect persisted after the completion or discontinuation of zoledronic acid), while denosumab had no offset effect. Second, the persistence rate of zoledronic acid was better than that of denosumab at the end of the first year (i.e., 100% for zoledronic acid versus 81% for denosumab, respectively), although the cumulative persistence rates were similar at the end of the second or third years (i.e., 52 and 36% for zoledronic acid, versus 55 and 37% for denosumab, for the second and third years, respectively). Third, the annual cost of zoledronic acid was less expensive than that of denosumab. In addition, alendronate was only initiated among the 37% who were persistent with denosumab at the end of 3 years. Due to the lower cumulative persistence rate of alendronate, only 10% were persistent with alendronate at the end of its 3-year treatment period (i.e., at the end of 6 years of sequential denosumab/alendronate). Alendronate, therefore, made a small contribution to both cost and effectiveness in this analysis.

In our base case analysis, we did not use the results of a small single-center Japanese study (*n* = 102 patients) showing much higher persistence with denosumab than was found in a previous meta-analysis [23], because this study seemed to lack generalizability and similar results were not available in a Japanese setting for zoledronic acid. However, we explored these results in a sensitivity analysis, and found that sequential denosumab/alendronate became cost-effective at ages 70 or 75 if we used these results. If we extrapolated similarly high persistence levels to zoledronic acid, for which no persistence data were available in a Japanese setting, zoledronic acid remained the preferred strategy. Since there is no fundamental reason to believe that the persistence rates with denosumab were high but those with zoledronic acid were low in Japan, it is more plausible that the persistence rates of both zoledronic acid and denosumab are similar in Japan, reinforcing conclusions from the base case analysis.

In a deterministic sensitivity analysis, results were especially sensitive to the change in zoledronic acid efficacy for reduction of vertebral fracture. If we incorporated less favorable values for the efficacy of zoledronic acid in reducing vertebral fractures, denosumab/alendronate became cost-effective at the predetermined willingness-to-pay threshold of ¥5 million per QALY at ages 75 and 80. Zoledronic acid has a residual effect, which further extended its efficacy (or lack thereof) over a longer time period. These factors, coupled with the high incidence of vertebral fractures, contributed to results being sensitive to the efficacy of zoledronic acid for reduction of clinical vertebral fracture.

Current evidence is insufficient to determine whether the elevated fracture risk after discontinuation of denosumab represents a quick reversal to the baseline pre-treatment risk (i.e., quick deterioration of the therapeutic effect) or a true rebound leading to an increase above the baseline pre-treatment risk [4]. In this analysis, we conservatively assumed that the elevated fracture risk represents a quick reversal to the baseline pre-treatment risk. If, however, we assumed the elevated fracture risk represents a rebound increase above the baseline pre-treatment risk, the results would be further in favor of zoledronic acid compared with sequential denosumab/alendronate.

In this study, the target population was postmenopausal osteoporotic women in Japan without a prior fragility fracture. As individuals with a previous fracture are at higher risk of a future fracture [43], we believe that zoledronic acid would remain cost-saving compared with sequential denosumab/alendronate for those osteoporotic women with a prior fragility fracture.

We note several limitations. First, although alendronate has been shown to maintain BMD after discontinuation of denosumab [44], sequential denosumab/bisphosphonate has not been rigorously evaluated with regard to how well it prevents actual fractures; to date, to the best of our knowledge, there has not been a published report of a randomized controlled trial in which the outcome was fracture prevention. Second, the persistence rates with zoledronic acid or denosumab were the critical parameters in this study. However, current data on these parameters in the Japanese setting were limited. Third, our model only included hip and clinical vertebral fractures, as data are limited regarding the costs of treatments and annual incidence rates of the other types of osteoporotic fractures such as distal forearm or proximal humerus fractures. However, we believe that hip and clinical vertebral fractures are likely to be the essential clinical events that need to be explicitly modeled and including the other types of fractures would have little influence on the overall results [13]*.* Fourth, although intravenous zoledronic acid after the completion of denosumab seems to be an alternative option, this approach was beyond the scope of our study [45]. We intended to focus on the initial treatment (i.e., zoledronic acid vs. denosumab) in this study. Finally, our results may be best applied to postmenopausal women in Japan and may not generalize to women of other races/ethnicities or in other countries, or men.

Despite these limitations, our study has notable strengths. First, to our knowledge, this is the first economic evaluation worldwide to compare the cost-effectiveness of zoledronic acid and sequential denosumab/alendronate to treat osteoporosis. We used a realistic scenario to incorporate recent evidence regarding denosumab (i.e., those treated with denosumab should not have a drug holiday after a given treatment period in contrast to those treated with bisphosphonates, and no offset effect was assumed after the completion of denosumab and subsequent alendronate was initiated). Second, we incorporated medication persistence and adherence into the model and extensively examined how these parameters’ changes affect the ICERs in deterministic sensitivity analyses, as persistence and adherence rates are known to be essential parameters in cost-effectiveness analyses regarding osteoporosis [2, 12, 13].

In conclusion, among hypothetical cohorts of community-dwelling older osteoporotic women without fragility fracture in Japan, annual intravenous zoledronic acid for 3 years was cost-saving (i.e., more effective and less expensive) compared with sequential biannual subcutaneous denosumab for 3 years followed by weekly oral alendronate for 3 years. This study provides practical and useful insights for clinicians and policymakers from the health economic perspective regarding osteoporosis treatment in older women in Japan.

## Supplementary Information

Below is the link to the electronic supplementary material.

Supplementary file1 The Consolidated Health Economic Evaluation Reporting Standards (CHEERS) statement (DOCX 22 KB)

Supplementary file2 (DOCX 16 KB)
