# Supplementary material for: Cost-effectiveness of zoledronic acid compared with sequential denosumab/alendronate for older osteoporotic women in Japan
Source: Arch Osteoporos. 2021 Jul 15;16(1):113. doi: 10.1007/s11657-021-00956-z (PMC8282566; doi:10.1007/s11657-021-00956-z)
Supplement: Supplementary file 1 — Supplementary file1 The Consolidated Health Economic Evaluation Reporting Standards (CHEERS) statement (DOCX 22 KB) [file 11657_2021_956_MOESM1_ESM.docx]

**Supplemental Table 1: CHEERS checklist—Items to include when reporting economic evaluations of health interventions**

| **Section/item** | **Item no.** | **Recommendation** | **Reported on**  **page no./line no.** |
| --- | --- | --- | --- |
| **Title and abstract** | | | |
| Title | 1 | Identify the study as an economic evaluation, or use more specific terms such as “cost-effectiveness analysis” and describe the interventions compared. | page 1, line 1- |
| Abstract | 2 | Provide a structured summary of objectives, perspective, setting, methods (including study design and inputs), results (including base-case and uncertainty analyses), and conclusions. | page 1, line 6- |
| **Introduction** | | | |
| Background and objectives | 3 | Provide an explicit statement of the broader context for the study. Present the study question and its relevance for health policy or practice decisions. | Page 2, left column, line 1- |
| **Methods** | | | |
| Target population and subgroups | 4 | Describe characteristics of the base-case population and subgroups analyzed including why they were chosen. | Page 2, right column, line 19- |
| Setting and location | 5 | State relevant aspects of the system(s) in which the decision(s) need(s) to be made. | Page 2, right column, line 19- |
| Study perspective | 6 | Describe the perspective of the study and relate this to the costs being evaluated. | Page 2, right column, line 39- |
| Comparators | 7 | Describe the interventions or strategies being compared and state why they were chosen. | Page 2, right column, line 5- |
| Time horizon | 8 | State the time horizon(s) over which costs and consequences are being evaluated and say why appropriate. | Page 2, right column, line 28- |
| Discount rate | 9 | Report the choice of discount rate(s) used for costs and outcomes and say why appropriate. | Page 3, left column, line 2- |
| Choice of health outcomes | 10 | Describe what outcomes were used as the measure(s) of benefit in the evaluation and their relevance for the type of analysis performed. | Page 7, right column, line 40- |
| Measurement of effectiveness | 11a | *Single study–based estimates*: Describe fully the design features of the single effectiveness study and why the single study was a sufficient source of clinical effectiveness data. | not applicable |
|  | 11b | *Synthesis-based estimates*: Describe fully the methods used for the identification of included studies and synthesis of clinical effectiveness data. | Page 3, right column, line 11- |
| Measurement and valuation of preference-based outcomes | 12 | If applicable, describe the population and methods used to elicit preferences for outcomes. | not applicable |
| Estimating resources and costs | 13a | *Single study–based economic evaluation*: Describe approaches used to estimate resource use associated with the alternative interventions. Describe primary or secondary research methods for valuing each resource item in terms of its unit cost. Describe any adjustments made to approximate to opportunity costs. | not applicable |
|  | 13b | *Model-based economic evaluation***:** Describe approaches and data sources used to estimate resource use associated with model health states. Describe primary or secondary research methods for valuing each resource item in terms of its unit cost. Describe any adjustments made to approximate to opportunity costs. | Page 8, left column, line 1- |
| Currency, price date, and conversion | 14 | Report the dates of the estimated resource quantities and unit costs. Describe methods for adjusting estimated unit costs to the year of reported costs if necessary. Describe methods for converting costs into a common currency base and the exchange rate. | Page 2, right column, line 25- |
| Choice of model | 15 | Describe and give reasons for the specific type of decision-analytic model used. Providing a figure to show model structure is strongly recommended. | Page 3, left column, line 21-, Fig.1 |
| Assumptions | 16 | Describe all structural or other assumptions underpinning the decision-analytic model. | Page 2, left column, line 17- |
| Analytic methods | 17 | Describe all analytic methods supporting the evaluation. This could include methods for dealing with skewed, missing, or censored data; extrapolation methods; methods for pooling data; approaches to validate or make adjustments (e.g., half-cycle corrections) to a model; and methods for handling population heterogeneity and uncertainty. | Page 2, left column, line 17- |
| **Results** | | | |
| Study parameters | 18 | Report the values, ranges, references, and if used, probability distributions for all parameters. Report reasons or sources for distributions used to represent uncertainty where appropriate. Providing a table to show the input values is strongly recommended. | Page 4, Table 2 |
| Incremental costs and outcomes | 19 | For each intervention, report mean values for the main categories of estimated costs and outcomes of interest, as well as mean differences between the comparator groups. If applicable, report incremental cost-effectiveness ratios. | Page 9, left column, line 31-, Table 3 |
| Characterizing uncertainty | 20a | *Single study–based economic evaluation*: Describe the effects of sampling uncertainty for estimated incremental cost, incremental effectiveness, and incremental cost-effectiveness, together with the impact of methodological assumptions (such as discount rate, study perspective). | not applicable |
|  | 20b | *Model-based economic evaluation*: Describe the effects on the results of uncertainty for all input parameters, and uncertainty related to the structure of the model and assumptions. | Page 9, right column, line 1-, Fig. 2, Table 4 |
| Characterizing heterogeneity | 21 | If applicable, report differences in costs, outcomes, or cost-effectiveness that can be explained by variations between subgroups of patients with different baseline characteristics or other observed variability in effects that are not reducible by more information. | not applicable |
| **Discussion** | | | |
| Study findings, limitations, generalizability, and current knowledge | 22 | Summarize key study findings and describe how they support the conclusions reached. Discuss limitations and the generalizability of the findings and how the findings fit with current knowledge. | Page 9, right column, line 38- |
| **Other** | | | |
| Source of funding | 23 | Describe how the study was funded and the role of the funder in the identification, design, conduct, and reporting of the analysis. Describe other nonmonetary sources of support. | Page 13, left column, line 10- |
| Conflicts of interest | 24 | Describe any potential for conflict of interest among study contributors in accordance with journal policy. In the absence of a journal policy, we recommend authors comply with International Committee of Medical Journal Editors’ recommendations. | Page 13, left column, line 13- |

**Supplemental Table 2: Osteoporosis-specific checklist: specific items to include when reporting economic evaluations on osteoporosis**

| **Item** | **Item no.** | **Recommendation** | **Reported on page no./line no.** |
| --- | --- | --- | --- |
| Transition probabilities | 1 | Report the transition probabilities and how they were estimated (including increased fracture risk) | Page 7, right column, line 15- |
| Excess mortality after fractures | 2 | Describe approaches and data sources used for the excess mortality after fractures | Page 7, right column, line 30- |
| Fractures costs | 3 | Describe approaches and data sources used for fractures costs | Page 8, right column, line 16- |
| Fractures effects on utility | 4 | Describe approaches and data sources used for the effects of fractures on utility | Page 7, right column, line 40- |
| Treatment effect during treatment | 5 | Describe fully the methods used for the identification, selection, and synthesis of clinical effectiveness data (per fracture site) | Page 3, right column, line 11- |
| Treatment effect after discontinuation | 6 | Describe fully the methods used for the treatment effect after discontinuation | Page 7, left column, line 48- |
| Medication adherence | 7 | Describe approaches and data sources used for modeling medication adherence | Page 7, left column, line 30- |
| Treatment costs | 8 | Describe approaches and data sources used for therapy costs | Page 8, left column, line 11- |
| Treatment side effects | 9 | Describe approaches and data sources used for costs and utilities effects of adverse events | Page 8, right column, line 9- |
