# Supplementary material for: Cost-effectiveness of zoledronic acid compared with sequential denosumab/alendronate for older osteoporotic women in Japan
Source: Arch Osteoporos. 2021 Jul 15;16(1):113. doi: 10.1007/s11657-021-00956-z (PMC8282566; doi:10.1007/s11657-021-00956-z)
Supplement: Supplementary file 2 — Supplementary file2 (DOCX 16 KB) [file 11657_2021_956_MOESM2_ESM.docx]

Osteoporosis-specific checklist: specific items to include when reporting economic evaluations on osteoporosis

| **Item** | **Item no.** | **Recommendation** | **Reported on page no./line no.** |
| --- | --- | --- | --- |
| Transition probabilities | 1 | Report the transition probabilities and how they were estimated (including increased fracture risk) | Page 12, line 22- |
| Excess mortality after fractures | 2 | Describe approaches and data sources used for the excess mortality after fractures | Page 13, line 5- |
| Fractures costs | 3 | Describe approaches and data sources used for fractures costs | Page 15, line 27- |
| Fractures effects on utility | 4 | Describe approaches and data sources used for the effects of fractures on utility | Page 13, line 32- |
| Treatment effect during treatment | 5 | Describe fully the methods used for the identification, selection, and synthesis of clinical effectiveness data (per fracture site) | Page 9, line 54- |
| Treatment effect after discontinuation | 6 | Describe fully the methods used for the treatment effect after discontinuation | Page 11, line 49- |
| Medication adherence | 7 | Describe approaches and data sources used for modeling medication adherence | Page 11, line 17- |
| Treatment costs | 8 | Describe approaches and data sources used for therapy costs | Page 14, line 2- |
| Treatment side effects | 9 | Describe approaches and data sources used for costs and utilities effects of adverse events | Page 15, line 17- |
